# Effects of Different Weak Small Organic Acids on Clofazimine Solubility in Aqueous Media

**DOI:** 10.3390/pharmaceutics16121545

**Published:** 2024-12-02

**Authors:** Igor A. Topalović, Olivera S. Marković, Miloš P. Pešić, Mufaddal H. Kathawala, Martin Kuentz, Alex Avdeef, Abu T. M. Serajuddin, Tatjana Ž. Verbić

**Affiliations:** 1 University of Belgrade—Faculty of Chemistry, Studentski trg 12–16, 11000 Belgrade, Serbia; itopalovic22@gmail.com (I.A.T.); mpesic@chem.bg.ac.rs (M.P.P.); 2 University of Belgrade—Institute of Chemistry, Technology and Metallurgy, Department of Chemistry, Njegoševa 12, 11000 Belgrade, Serbia; olivera.markovic@ihtm.bg.ac.rs; 3 College of Pharmacy and Health Sciences, St. John’s University, Queens, New York, NY 11439, USA; mufaddal.kathawala17@my.stjohns.edu; 4 School of Life Sciences FHNW, Institute for Pharma Technology, University of Applied Sciences and Arts Northwestern Switzerland, Hofackerstraße, 30, 4132 Müttenz, Switzerland; martin.kuentz@fhnw.ch; 5 In-ADME Research, New York, NY 10128, USA; alex@in-adme.com

**Keywords:** clofazimine, acid–base supersolubilization (ABS), solubility, glutaric acid, tartaric acid, malic acid, *shake-flask* method

## Abstract

Background/Objectives: Clofazimine (CFZ) is a Biopharmaceutics Classification System (BCS) II drug introduced in the US market in 1986 for the treatment of leprosy. However, CFZ was later withdrawn from the market due to its extremely low aqueous solubility and low absorption. In the literature, the intrinsic solubility of CFZ has been estimated to be <0.01 μg/mL, and solubilities of its different salt forms in simulated gastric and intestinal fluids are <10 µg/mL. These are extremely low solubilities for the dissolution of a drug administered orally at 100–200 mg doses. Methods: In the present investigation, seven weak organic acids (adipic, citric, glutaric, maleic, malic, succinic, and tartaric) were tested by determining the aqueous solubility of CFZ as the function of acid concentration to investigate whether any of the acids would lead to the supersolubilization of CFZ. Results: There were only minimal increases in solubilities when concentrations of acids in water were increased up to 2.4 M. The solubilities, however, increased to 0.32, 1.23, and 10.68 mg/mL, respectively, in 5 M solutions of tartaric, malic, and glutaric acids after equilibration for 24 h at 25 °C. Crystalline solids were formed after the equilibration of CFZ with all acids. Apparently, salts or cocrystals were formed with all acids, except for glutaric acid, as their melting endotherms in DSC scans were in the range of 207.6 to 248.5 °C, which were close to that of CFZ itself (224.8 °C). In contrast, the adduct formed with glutaric acid melted at the low temperature of 77 °C, and no other peak was observed at a higher temperature, indicating that the material converted to an amorphous state. Conclusions: The increase in CFZ solubility to >10 mg/mL in the presence of 5 M glutaric acid could be called supersolubilization when compared to the intrinsic solubility of the basic drug. Such an increase in CFZ solubility and the conversion of the glutarate adduct to an amorphous state are being exploited to develop rapidly dissolving dosage forms.

## 1. Introduction

**Figure 1:**
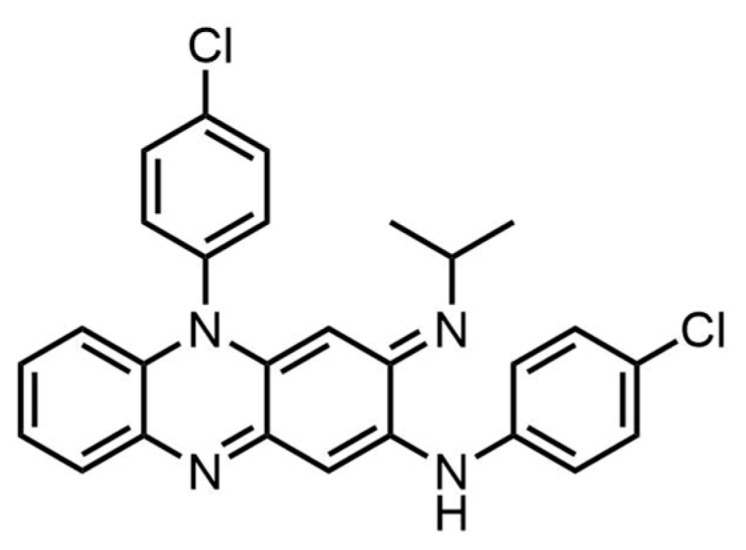
Structure of clofazimine (CFZ).

Clofazimine (CFZ, Figure 1), which was discovered in 1957 [1], is an antibiotic and an anti-inflammatory drug that was used therapeutically to treat leprosy (Hansen’s disease) [2]. It was also repurposed as an anti-tuberculosis (TB) drug for treating *Mycobacterium tuberculosis* diseases, especially those resistant to other drugs [3,4,5]. Additionally, CFZ showed inhibitory activity against disseminated *Mycobacterium avium* complex disease in HIV-infected patients [6] and against coronaviruses [7]. It has also been reported that CFZ may be effective for treating cryptosporidiosis, a parasitic disease caused by *Cryptosporidium* spp., which includes prolonged episodes of watery diarrhea along with other untoward symptoms [8,9].

TB is one of the deadliest infectious diseases in the world. According to the 2023 World Health Organization (WHO) Global Tuberculosis Report [10], TB was the world’s second leading cause of death in 2022 from a single infectious agent, only after coronavirus disease (COVID-19), and it caused almost twice as many deaths as HIV/AIDS. The same report also mentions that there were over 10 million people infected with TB in 2022, with 7.5 million new cases and 1.3 million deaths. However, according to this WHO report, the treatment success rate for people infected with tuberculosis using different drugs is somewhat limited; for drug-susceptible TB, it is 88%, and for drug-resistant TB (MDR/RR-TB), it is only 63%. This situation is unfavorable to the United Nations (UN) goal of ending TB by 2030. Better treatment, especially for drug-resistant TB, is needed. Therefore, the use of CFZ against tuberculosis (TB) is particularly noteworthy. The effect of clofazimine in drug-resistant TB was previously reviewed [11]. More recently, Wang et al. [12] concluded that the drug has the potential to achieve better outcomes, treatment completion, and cure rates, as well as a lower treatment failure risk in drug-resistant TB than in patients who do not receive clofazimine. 

CFZ was originally marketed by Novartis in 1986 as a micronized drug suspended in an oil–wax base with the trade name Lamprene. However, because of the extremely poor aqueous solubility of CFZ, the product faced many pharmacokinetic, bioavailability, and therapeutic issues [13,14,15,16], and, therefore, the company no longer markets the product in the USA. No other generic or brand-name equivalents of CFZ are also available in the USA. Similarly, despite being included in the WHO essential drug list, there are no CFZ products in the global market that provide optimal therapy. 

CFZ is a Biopharmaceutics Classification System (BCS) class II drug with a reported water solubility of <0.01 μg/mL and a log*P* of 7.7 [17]. According to another report, the solubility of CFZ in water ranges from 0.49 to 1.03 μg/mL, and its intrinsic solubility is about 0.02–0.03 μg/mL [4]. There are numerous approaches for solubility and dissolution enhancement of poorly water-soluble drugs reported in the literature: particle size reduction, solid dispersion, salt formation, conversion to an amorphous form, micellar solubilization, and nano-solubilization, among others, have been reported in the literature [18,19,20,21,22,23,24,25,26,27,28]. No such techniques could yet resolve formulation issues with CFZ.

Previously, Bolla and Nangia prepared CFZ salts with methanesulfonic acid, maleic acid, isonicotinic acid, nicotinic acid, malonic acid, and salicylic acid [5]. However, the aqueous solubilities of the salts formed were so low that the authors could not reliably determine their solubilities in water. The authors, therefore, resorted to determining solubility by dispersing powders in a 60% ethanol–water mixture. Even at such a high concentration of alcohol (60%), the solubilities of the salts were very low. The methanesulfonic acid provided the highest solubility at about 120 µg/mL in the 60% ethanol–water mixture at 37 °C, and the solubility of most other salts was much lower. In another report, Bannigan et al. [17] conducted dissolution testing of eight different CFZ salts, namely, phosphate, sulfate, acetate, formate, citrate, oxalate, nitrate, and hydrochloride in aqueous media like water, fasted-state simulated gastric fluid (FaSSGF), and fasted-state simulated intestinal fluid (FaSSIF), and observed the apparent solubilities of the salts, as determined by maximum concentrations in dissolution media, to be very low. For example, the apparent solubilities of the salts were <10 µg/mL after 20 min of equilibration in FaSSGF, while in FaSSIF, the highest solubility in any salt observed was about 10 µg/mL after 10 min, which then decreased to ~5 µg/mL after 60 min. The solubilities of the salts were very low despite the presence of physiological surfactants like bile salt and lecithin in simulated gastric and intestinal fluids. 

Because of the extremely low aqueous solubility of CFZ and its relatively high oral dose of 100–200 mg, we have undertaken systematic studies on the characterization of the physicochemical properties of the drug and the development of a viable dosage form for it. In a previous report, Verbić et al. [29] reported on the p*K*_a_ determination of CFZ, where a unique cosolvent dependence of p*K*_a_ was revealed along with the formation of a CFZ free-base dimer in alkaline solutions. The aqueous p*K*_a_ value of CFZ at 25 °C (0.15 M reference ionic strength) was extrapolated to a zero cosolvent in methanol−water media using potentiometric (p*K*_a_ 9.43) and UV/vis spectrophotometric (p*K*_a_ 9.61) titrations [29].

The present investigation aims to study whether the solubility of CFZ in aqueous media could be increased using a novel technique called acid–base supersolubilization (ABS), which is reported in the literature [30,31,32,33,34]. The ABS principle has been developed based on the classical pH vs. solubility theory [35,36], where the solubility of weakly basic drugs increases when acids are used to reduce the pH of their aqueous solutions. Salts may crystallize when the pH is lowered below pH_max_ or the pH of maximum solubility in water. However, if the acids used are weak, such that they do not lower pH below pH_max_, no salts are formed, and, instead, the drug solubility keeps increasing until a very high solubility is reached. This is the basis of the ABS principle to increase the solubility of weakly basic drugs. A similar principle also applies to acidic drugs, where the solubility can be increased greatly by adjusting the pH with weak bases [34]. 

In the present investigation, seven relatively weak organic acids, namely, citric, succinic, malic, tartaric, maleic, glutaric, and adipic acids, were selected for testing whether the acid–base supersolubilization (ABS) effect could be observed with any of the acids in an aqueous medium. No organic solvent was added to the medium. Structures and p*K*_a_ values of the acids used are shown in Table 1. CFZ solubility was determined using the *shake-flask* method, following the recommendations published in a *white paper* [37]. The effects of acid concentration, the solution pH value, and the equilibration time on the CFZ solubility were also investigated. This type of research can be valuable for formulating such an important drug into viable dosage forms. 

## 2. Materials and Methods

### 2.1. Chemicals and Reagents

The chemicals used in this study were purchased from the following sources: clofazimine (AK Scientific, Union City, CA, USA, CAS number: 2030-63-9, purity: 98% (HPLC)), glutaric acid (Acros organics, Geel, Belgium, CAS number: 110-94-1, purity: 99.8% (Titration with NaOH)), citric acid monohydrate (Kemika, Zagreb, Croatia, CAS number: 5949-29-1, purity: analytical grade), malic acid (Merck KGaA, Darmstadt, Germany, CAS number: 6915-15-7, purity: analytical standard), tartaric acid (Carlo Erba Reagents GmbH, Emmendingen, Germany, CAS number: 87-69-4, purity: 99.7–100.5 (assay acidimetric)), maleic acid (Kemika, Zagreb, Croatia, CAS number: 110-16-7, purity: analytical grade), succinic acid (Merck KGaA, Darmstadt, Germany, CAS number; 110-15-6, purity: ≥99.0%), adipic acid (Kemika, Zagreb, Croatia, CAS number: 124-04-9, purity: analytical grade), hydrochloric acid and sodium hydroxide (Titrisol ampoules, Merck KGaA, Darmstadt, Germany, CAS numbers: 7647-01-0, 1310-73-2, purity: analytical grade), phosphoric acid (Fluka, Honeywell International Inc, Seelze, Germany, CAS number: 7664-38-2, purity: HPLC grade), and sodium dihydrogen phosphate (Fluka, Honeywell International Inc, Seelze, Germany, CAS number: 7558-80-7, purity: HPLC grade). Deionized (DI) water was used to prepare all aqueous solutions.

### 2.2. pH Measurement

Values of pH were measured using *p*SOL Gemini Profiler (*p*ION Inc., Billerica, MA, USA) equipped with a Thermo Scientific Orion PerpHecT ROSS microelectrode (PN 8220BNWP, Waltham, MA, USA). The electrode was calibrated using a Radiometer (Copenhagen, Denmark) standard buffer solution (pH 7.00). The operational pH meter values were converted to values based on the concentration scale (p[H^+^] = −log[H^+^]), employing an HCl−NaOH blank titration using the Avdeef–Bucher equation [39].

### 2.3. HPLC Analysis

The drug concentration in the supernatant solutions was determined using an HPLC-UV/Vis system (Agilent Technologies 1260 Infinity LC System) with Zorbax Eclipse XDB–C18 4.6 × 50 mm^2^ column packed with 1.8 μm particles. Chromatographic analysis was performed at 25 °C column temperature using isocratic elution. Other HPLC conditions include the mobile phase: a 45 to 55 *V*/*V* mixture of 0.1 M aqueous phosphate buffer (pH = 3.1) and acetonitrile; injection volume: 2 or 10 μL; flow rate: 1 mL/min; detection wavelength: 284 nm. The aliquots of drug solutions were appropriately diluted with acetonitrile before being injected into HPLC. Chromatograms of CFZ external standard solutions and corresponding calibration diagrams are shown in Figures S1 and S2 in the Supporting Information.

### 2.4. Solubility Determination Using the Shake-Flask Method

#### 2.4.1. Screening of Different Weak Organic Acids for Clofazimine Solubility

Different acids were screened for their solubilizing effects on clofazimine. For citric, malic, tartaric, maleic, or glutaric acids, 2.4 M acid solutions were prepared, and then CFZ was equilibrated in the solutions. For this purpose, an accurately weighed amount of CFZ was added to 1.0 mL of each acid solution and then equilibrated by the *shake-flask* method for 1 h. The amounts of CFZ added to 1.0 mL of each solution ranged from 8 to 11 mg, except for glutaric acid, where 16.67 mg of CFZ was added. The relatively short equilibration period of 1 h was used because the interaction between the acid and base that leads to acid–base supersolubilization usually occurs almost immediately [30]. The equilibration time was later extended for selected acids to determine the effect of time on the solubility of CFZ. After equilibration, the aqueous phase from each suspension was separated from the solid phase by filtration through a hydrophobic 0.22 µm PVDF filter. HPLC analysis of filtrates was conducted for drug concentrations according to the method described earlier. 

CFZ solubility in succinic and adipic acid could not be determined at 2.4 M acid concentrations due to the low aqueous solubility (<1 M) of these acids. For them, a 0.14 M adipic acid solution and 0.64 M succinic acid solution were used, and 3–5 mg of CFZ was equilibrated per mL of each solution. The rest of the procedure for determining drug solubility remained the same.

#### 2.4.2. The Effect of Acid Concentration on CFZ Solubility

Based on the results of screening seven weak organic acids for CFZ solubility mentioned earlier, tartaric, malic, and glutaric acid were selected for further studies, where the effect of the acid concentration on CFZ solubility was first determined. Solutions of different acids in the concentration range of 0.01 to 5 M were used; although the actual concentrations of different acids varied slightly, they were around 0.010, 0.015, 0.05, 0.2, 0.5, 1.0, 2.0, 3.0, 4.0, 4.5, and 5.0 M. The amount of CFZ added to 1 mL of each solution was about 15 mg, except for glutaric acid, which ranged from 15 to 77 mg. The suspensions were equilibrated for 6 h, followed by 18 h of sedimentation. The supernatant liquids were filtered through 0.22 µm PVDF filters before HPLC analysis.

#### 2.4.3. The Effect of Equilibration Time on CFZ Solubility

To determine the effect of equilibration time on CFZ solubility, 2 M and 5 M solutions of tartaric acid, malic acid, and glutaric acid in water were prepared, and approximately 15 mg of CFZ was added to 1 mL of each solution, except for GA, where the amount of added drug ranged from 15 to 50 mg. The suspensions were equilibrated for different times from 1 to 24 h and then filtered through 0.22 µm PVDF filters for HPLC analysis of the filtrates.

### 2.5. Solid State Characterization of Solid Phases in Equilibrium with Acid Solutions

#### 2.5.1. Isolation of Solid Materials

For the isolation of solids formed by the interaction of CFZ with different acids, 20 mL of each acid solution in water was prepared; the concentration of acids was ~5 M for glutaric, malic, and tartaric acids, ~2.4 M for citric and maleic acids, and 0.64 M and 0.14 M, respectively, for succinic and adipic acids. A small excess of solid CFZ was added to 20 mL of each acid solution and then equilibrated by shaking in a water bath for 6 h using a wrist action shaker, followed by 18 h of settling. After equilibration, the solids were isolated by vacuum filtration, washed once with water, and dried overnight under vacuum at 40 °C. Finally, the dried materials were analyzed using differential scanning calorimetry (DSC) and powder X-ray diffraction (PXRD).

#### 2.5.2. Differential Scanning Calorimetric (DSC) Analysis

The DSC analysis of samples was performed using a Q200 modulated DSC analyzer (TA Instruments, New Castle, DE, USA) by placing 5–10 mg of each material in a Tzero aluminum pan and sealing it hermetically. To facilitate the escape of any volatile material produced, a pinhole was dented in the lid of the pan. After equilibrating at 25 °C, the samples were heated at a ramp rate of 5 °C per minute until a temperature of 300 °C was attained.

#### 2.5.3. Powder X-Ray Diffraction (PXRD)

A Shimadzu XRD-6000 diffractometer (Shimadzu, Kyoto, Japan) equipped with a Ni filter and monochromatic Cu-Kα radiation source was used for PXRD analysis. It was operated at a current of 30 mA with a copper anode tube at a generator voltage of 40 kV, and the samples were scanned at a rate of 2°Ɵ/min across the scan range of 5–40°Ɵ.

### 2.6. Molecular Dynamic (MD) Simulations

Molecular dynamic (MD) simulations were based on the YASARA software version 20.12.24 (YASARA Biosciences GmbH, Vienna, Austria) [40]. Accelerated calculations were obtained by the use of a graphic processing unit (GPU) for the estimation of the non-bonded interactions (Van der Waals and real-space Coulomb forces [38]). A general AMBER-type force field, GAFF2, was employed [41], with atomic charges based on a semi-empirical quantum chemical estimation (AM1BCC) [42]. At the same time, the protonation state of CFZ was verified as major microspecies using the software Marvin v.19.3. (ChemAxon Ltd., Budapest, Hungary). Four molecules of CFZ and a co-former (either glutaric or succinic acid) of about 1 M concentration were randomly placed in a simulation box of the dimension 65 × 65 × 65 Å, which was subsequently filled with TIP3P-rigid water molecules. Periodic boundary conditions were set, and following steepest decent and simulated annealing minimizations to remove clashes, simulations were run as *n* = 4 replicates for 10 ns equilibration time at 298 K. Equations of motion were integrated with a 2 × 1 fs time step as an NTP ensemble, where the pressure was controlled by rescaling the simulation cell along the *x*, *y*, and *z*-axis to reach a constant pressure of 1 bar. A cut-off value of 8 Å was selected for the Van der Waals forces, and the Particle Mesh Ewald method was used for efficient long-range electrostatic calculations [43]. Following the main equilibration cycle, an estimation of the mean interaction forces of CFZ and an additive was based on a configurational sampling in an additional 200 ps production cycle.

## 3. Results and Discussion

### 3.1. Screening of Weak Organic Acids for CFZ Solubility

The concentrations of CFZ solutions in water with all studied organic acids after a 1 h shake-flask equilibration are given in Table 2. For citric, glutaric, malic, maleic, and tartaric acids, concentrations of ~2.4 M were used, and the concentrations of adipic and succinic acids were as high as possible but much lower than 2.4 M due to their lower solubility in water. The exact concentrations of the acids used, pH values of solutions after equilibration with CFZ, and solubilities of CFZ expressed in a molar concentration and as mg/mL are given in Table 2.

As shown in Table 2, after 1 h of *shake-flask* equilibration, only malic, tartaric, and glutaric acids showed significantly high solubilities in the range of 0.16 to 0.62 mg/mL, which are much higher than those observed by Bolla and Nangia [5] and Bannigan et al. [17] for any of the 14 salts they studied, as mentioned earlier. The acids used in the present investigation were selected for further studies to determine their effects on CFZ solubility.

### 3.2. Effect of Acid Concentration on CFZ Solubility

**Figure 2:**
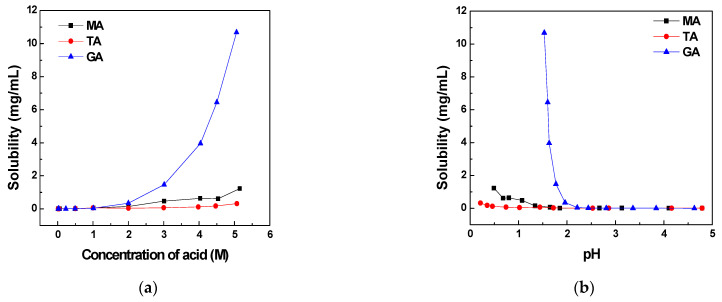
Effect of acid concentration (**a**) and solution pH (**b**) on CFZ solubility in malic (MA), tartaric (TA), and glutaric acid (GA) solutions at 25 °C.

The solubilities of CFZ with increasing concentrations of malic, tartaric, and glutaric acids are shown in Figure 2, where, in Figure 2a, solubilities are plotted as the function of acid concentration, and, in 2b, they are plotted as the function of pH observed after the equilibration of solutions. Since the solubility values in Figure 2 are very low and cannot be easily differentiated from the lines in the figure, they are also tabulated in Table 3, Table 4 and Table 5, where the exact concentrations of acids used, the pH before and after equilibration with CFZ, and concentrations of CFZ in both molar and mg/mL units are given.

These results show that tartaric acid and malic acid had very low solubilizing effects on CFZ as the solubilities were extremely low at acid concentrations of 0.01 M to 1.0 M. Even at the acid concentration of 5 M, solubilities in tartaric and malic acids increased only to 0.32 and 1.2 mg/mL, respectively. Although the solubility of CFZ was also low at a low glutaric acid concentration, it increased sharply when the acid concentration was raised gradually from 1 M to 5 M. For example, while the CFZ solubility was 0.045 mg/mL in 1 M glutaric acid, it increased to 10.7 mg/mL in 5 M glutaric acid. It may be noted that the solubilizing effects of glutaric acid increased greatly in its highly concentrated aqueous solutions. While the solubility of CFZ in 4 M glutaric acid was 4 mg/mL, it increased almost three times to 10.7 mg/mL when the acid concentration increased by only one molar concentration from 4 to 5 M. As compared to zero or undetectable solubility of CFZ in water, the increase in solubility to 10.7 mg/mL in 5 M glutaric acid may be considered a supersolubilization. As presented later, the MD simulations revealed that the special interaction between CFZ and glutaric acid in the solution to form clusters could be responsible for the supersolubilizing effect observed.

### 3.3. Effect of Equilibration Time on CFZ Solubility

**Figure 3:**
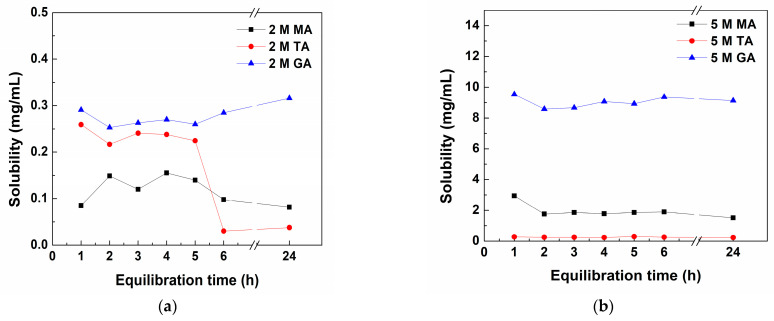
Effect of equilibration time on CFZ solubility in 2 M (**a**) and 5 M (**b**) malic (MA), tartaric (TA), and glutaric acid (GA) solutions at 25 °C.

The effect of equilibration time on CFZ solubility in 2 M and 5 M solutions of glutaric, malic, and tartaric acids with increasing stirring times from 1 to 6 h (1, 2, 3, 4, 5, and 6 h) and a stirring time for 6 h following 18 h of sedimentation (total 24 h) was studied. The results are shown in Figure 3 and Tables S1–S3 in the Supporting Information.

The above results demonstrated that the equilibration of CFZ in different acid solutions is rapid, as there is no significant difference in its solubility between 1 and 24 h equilibration, except for the 2 M tartaric acid, where the solubility dropped, possibly due to the crystallization of salt or cocrystals. Based on these results, it may be concluded that all solubility data plotted in Figure 2 and Table 3, Table 4 and Table 5 are equilibrium values, as they were determined by the equilibration of suspensions for 6 h of equilibration (shaking), followed by 18 h of standing. 

### 3.4. Solid State Characterization of Materials Produced After Equilibration with Different Acids

**Figure 4:**
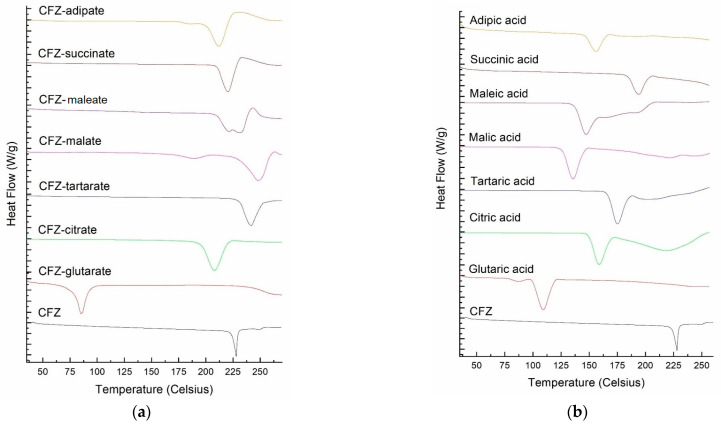
(**a**) DSC scans of new crystalline material formed when CFZ interacts with various weak acids; (**b**) DSC scans of the corresponding weak acids.

**Figure 5:**
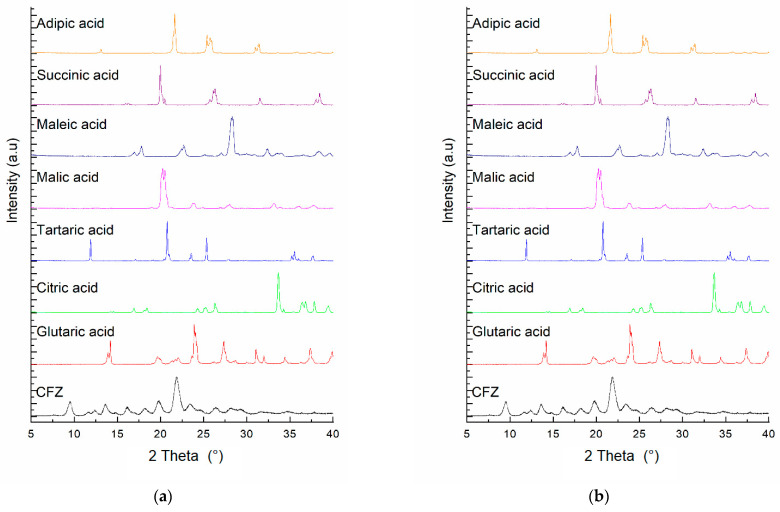
(**a**) PXRD pattern of the new crystalline solid formed when CFZ interacted with various weak acids; (**b**) PXRD pattern of the corresponding weak acids.

Solids obtained after the equilibration of CFZ as suspensions with different acids were characterized by DSC and PXRD, and the results are presented in Figure 4 and Figure 5. The DSC scans of the crystalline materials produced are shown in Figure 4a, and the DSC scans of the corresponding weak acids are provided in Figure 4b. The melting temperature of the new crystalline solids and the corresponding weak acids are tabulated in Table 6. These results demonstrate that all the materials in equilibria with acid solutions are crystalline, having distinct DSC endothermic peaks different from those of CFZ and individual acids. These CFZ-acid adducts could be either salts or cocrystals of CFZ, and there could be even amorphous CFZ mixed with excess crystalline acids. However, no studies to determine the crystal structures of the materials were conducted in the present investigation. 

Figure 4 and Table 6 demonstrate that the melting points of all the adducts formed, except for the one with glutaric acid, were in the range of 207.6 to 248.5 °C and comparable to that of the clofazimine base (224.8 °C). Thus, CFZ salts or cocrystals appeared to be formed with adipic, citric, maleic, malic, succinic, and tartaric acids. In contrast, the melting point of the adduct formed with glutaric acid was much lower at 77 °C. It was even lower than the melting point of glutaric acid itself (99.8 °C). There was no additional melting peak in the DSC scan of the CFZ-glutaric acid adduct at any temperature above 77 °C. Thus, it is evident that the material converted to an amorphous state upon heating above 77 °C, which may be attributed to the interaction between CFZ and glutaric acid. It is possible that such an interaction between the two components could also occur in aqueous media, leading to the supersolubilization effect observed.

Like the DSC results, the PXRD patterns of solid materials isolated from the CFZ suspensions in different acids, as shown in Figure 5, also confirm the conversion of CFZ into crystalline adducts. The diffraction patterns of the solids in Figure 5a are different from those of the acids themselves in Figure 5b.

### 3.5. MD Simulations

**Figure 6:**
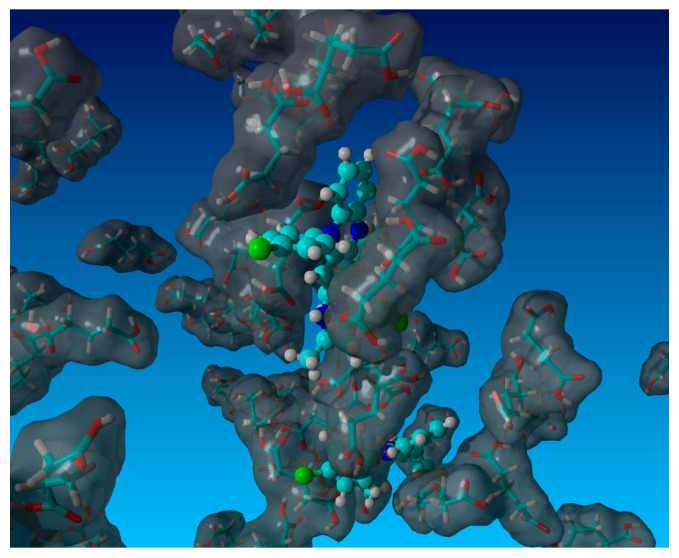
MD simulation snapshot (10 ns, 298 K, 1 bar) of CFZ (ball and stick model) and glutaric acid (tube model with Van der Waals molecular surface in gray) as co-former (~1 M concentration) with hidden TIP3P water. Details are given in the text.

**Figure 7:**
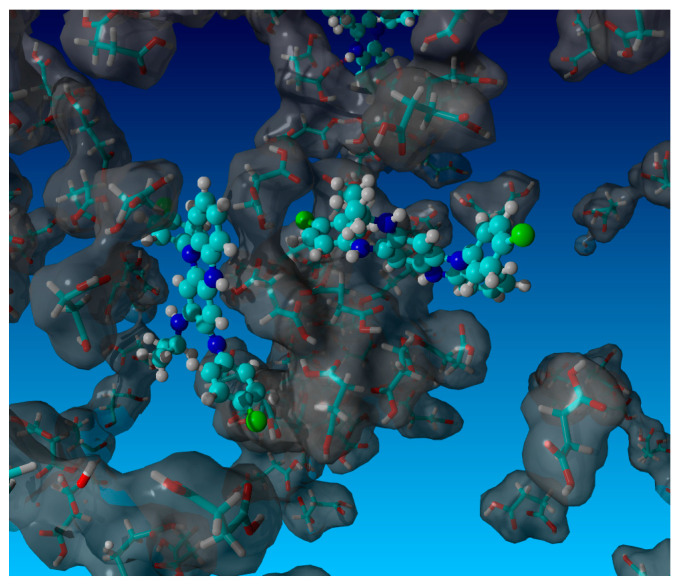
MD simulation snapshot (10 ns, 298 K, 1 bar) of CFZ (ball and stick model) and succinic acid (tube model with Van der Waals molecular surface in gray) as co-former (~1 M concentration), with hidden TIP3P water. Details are given in the text.

The molecular simulations targeted a mechanistic understanding of configurational aspects of the supersolubilization phenomenon. Section 3.2 indicated substantial differences in CFZ solubilization between the different co-formers as well as depending on their concentration. An important mechanism was apparently the ability of a specific co-former to reach comparatively high bulk concentrations without phase separating and crystallization. Glutaric acid provided the highest solubilization of CFZ, while, by comparison, succinic acid was one of the poorest solubilizing co-formers of CFZ, although it is a structurally similar bi-carboxylic acid with just one carbon less in the unbranched chain compared to glutaric acid. The digital comparison was made on a reference concentration of about 1 M of co-former, and Figure 6 (for glutaric acid) and Figure 7 (for succinic acid) show MD simulation snapshots (10 ns at 298 K, 1 bar) with a ball and stick model for CFZ and the tube model for the co-former, whereas water is not shown for the clarity of presentation. In all MD simulations, the co-former molecules revealed some clustering around the CFZ solute. This supramolecular phenomenon can be seen as a partial solvation layer, which did not entirely embed the solute but allowed the favorable exertion of hydrogen bonding and enabled contacts for Van der Waals interactions with CFZ. It appeared on a qualitative level that this supramolecular phenomenon was leading to fewer interactions between the co-former and the drug in the case of succinic acid compared to glutaric acid. The conformational sampling of the simulation production runs (*n* = 4) resulted in a CFZ and co-former hydrogen bonding interaction energy of 12.7 kJ/mol in the case of glutaric acid, which was significantly greater than the 6.9 kJ/mol of the hydrogen bonding interaction between the drug and succinic acid (*p* = 0.021; two sample *t*-test). The interaction energy in terms of hydrophobicity was overall dominant over hydrogen bonding, with 22.5 kJ/mol for CFZ and glutaric acid vs. 15.8 kJ/mol for the hydrophobic interaction energy of CFZ and succinic acid (*p* = 0.028). This result supported the initial qualitative view of the simulation snapshots in that glutaric acid provided a more effective partial solvation layer around CFZ than succinic acid. Moreover, the numerous contact points between CFZ and the co-former were leading to a net hydrophobic interaction energy that was greater than hydrogen bonding, even though the latter is, per contact point, by far the higher energetic interaction. It can be expected that at even higher concentrations than 1 M, the co-formers could cluster to an even greater extent around a poorly water-soluble solute like CFZ, but a direct in silico comparison with at least succinic acid would not be physically meaningful at concentrations much higher than the solubility of this bi-carboxylic acid. In summary, the MD simulations revealed interesting aspects of how a co-former can cluster around a poorly water-soluble solute. The molecular configurations and their associated interaction energies can either contribute to supersolubilization or alternatively may template for an undesirable phase separation/crystallization.

## 4. Conclusions

Clofazimine (CFZ) is an anti-leprosy and anti-TB drug with an estimated intrinsic solubility of <0.01 μg/mL, and among various salts, the highest solubility of ~120 µg/mL was observed for its mesylate salt in the 60% ethanol–water mixture at 37 °C. The solubilities of different salts in fasted-state simulated gastric and intestinal fluids during dissolution testing were reported to be <10 μg/mL and <6 μg/mL, respectively. Such solubilities are extremely low for a drug commonly administered orally at doses of 100–200 mg, which is responsible for the low and erratic bioavailability of the compound. We have determined CFZ solubility in seven weak organic acids (adipic, citric, glutaric, maleic, malic, succinic, and tartaric) as the function of acid concentration to investigate whether any supersolubilization effect could be observed with any of the acids. 

Preliminary studies have shown that only three of these acids, namely tartaric, malic, and glutaric, have some solubilizing effect on CFZ, so they were selected for further studies. However, any increase in CFZ solubility in the presence of tartaric and malic acids was relatively low. Even at the acid concentration of 5 M, solubilities in tartaric and malic acids increased only to 0.32 and 1.2 mg/mL, respectively. With glutaric acid, CFZ solubility increased sharply when the acid concentration was raised gradually from 1 M (0.045 mg/mL) to 5 M (10.7 mg/mL). Interestingly, the solubility increased close to three-fold from 4.0 to 10.7 mg/mL when the glutaric acid concentration increased by only one molar concentration, from 4 to 5 M, demonstrating that the concentrated solution of glutaric acid had a very high solubilizing effect. As compared to zero or undetectable solubility of CFZ in water, the increase in solubility to 10.7 mg/mL in 5 M glutaric acid may be considered a supersolubilization.

For molecular dynamics simulation studies, glutaric and succinic acids were selected; glutaric acid was identified as the best case in terms of solubilization of CFZ and succinic acid for comparison, as it was one of the poorest solubilizing co-formers of CFZ. In all MD simulations, the co-former molecules revealed some clustering around the CFZ solute. It appeared on a qualitative level that this supramolecular phenomenon was leading to fewer interactions for the co-former with the drug in the case of succinic acid compared to glutaric acid. 

These findings might be of paramount importance for formulating clofazimine, an important multipurpose drug, into viable dosage forms.

## Figures and Tables

**Table 1:** Structures and p*K*_a_ values of weak organic acids studied (25 °C, *I* = 0.15 M) ^1^.

Acid	Structure	p*K*_a_ ± SD	Acid	Structure	p*K*_a_ ± SD
Citric	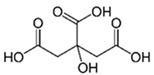	2.91 ± 0.01 4.34 ± 0.01 5.68 ± 0.02	Succinic	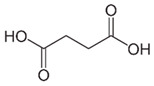	3.99 ± 0.01 5.21 ± 0.01
			Malic	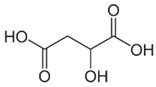	3.25 ± 0.01 4.68 ± 0.01
L-(+)-tartaric	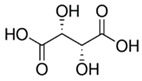	2.79 ± 0.01 3.90 ± 0.01	Maleic	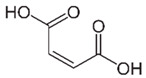	1.74 ± 0.01 5.81 ± 0.01
Glutaric	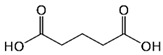	4.12 ± 0.07 5.02 ± 0.14	Adipic	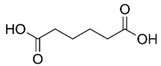	4.30 ± 0.06 5.15 ± 0.09

^1^ Deduced from multiple literature sources [38].

**Table 2:** Clofazimine (CFZ) solubility in ~2.4 M solutions of citric, glutaric, maleic, malic and tartaric acid, 0.14 M adipic, and 0.64 M succinic acid after 1 h equilibration at 25 °C with standard deviations obtained from three measurements.

Acids Used	Acid Concentration (M)	pH	Added CFZ Mass (mg)	CFZ Solubility (M)	CFZ Solubility (mg/mL)
Adipic	0.14	2.8	8.55	(1.1 ± 0.2) × 10^−5^	(5.0 ± 0.9) × 10^−3^
Citric	2.24	0.8	9.17	(7.2 ± 0.4) × 10^−5^	(3.4 ± 0.2) × 10^−2^
Glutaric	2.50	1.8	16.67	(1.3± 0.1) × 10^−3^	0.62 ± 0.05
Maleic	2.43	0.6	11.23	(1.53 ± 0.04) × 10^−5^	(7.2 ± 0.2) × 10^−3^
Malic	2.40	1.2	9.14	(3.3 ± 0.8) × 10^−4^	0.16 ± 0.04
Succinic	0.64	2.2	8.34	(4.05 ± 0.06) × 10^−5^	(1.92 ± 0.03) × 10^−2^
Tartaric	2.38	0.9	9.49	(9.7 ± 0.4) × 10^−4^	0.46 ± 0.02

**Table 3:** CFZ solubility with increasing concentrations of tartaric acid in water at 25 °C. Experimental details, pH values, and solubilities expressed as molar and mg/mL concentrations are also given with standard deviations obtained from three measurements.

Vial Number	Acid Concentration (M)	Volume Used (mL)	CFZ Added (mg)	pH			CFZ Solubility (M)	CFZ Solubility (mg/mL)
				Before CFZ Addition	Before Phase Separation	After Phase Separation		
1	1.01 × 10^−2^	1.002	14.70	3.9	4.5	4.8	(1.22 ± 0.08) × 10^−5^	(5.8 ± 0.4) × 10^−3^
2	1.53 × 10^−2^	1.003	14.54	3.5	4.1	4.2	(1.01 ± 0.09) × 10^−5^	(4.8 ± 0.5) × 10^−3^
3	2.17 × 10^−2^	1.004	14.63	3.1	2.9	2.9	(1.95 ± 0.06) × 10^−5^	(9.2 ± 0.3) × 10^−3^
4	3.57 × 10^−2^	1.005	14.62	2.6	2.5	2.5	(2.3 ± 0.1) × 10^−5^	(1.1 ± 0.5) × 10^−2^
5	0.500	1.000	14.86	1.7	1.6	1.7	(4.9 ± 0.5) × 10^−5^	(2.3 ± 0.3) × 10^−2^
6	1.00	1.000	15.02	1.4	1.4	1.4	(1.3 ± 0.2) × 10^−4^	(6.3 ± 0.9) × 10^−2^
7	2.00	1.000	15.43	1.1	1.0	1.0	(8.8 ± 0.5) × 10^−5^	(4.2 ± 0.3) × 10^−2^
8	3.00	1.000	14.80	0.7	0.6	0.7	(1.5 ± 0.1) × 10^−4^	(7.3 ± 0.5) × 10^−2^
9	3.98	1.000	15.00	0.4	0.4	0.5	(2.5 ± 0.1) × 10^−4^	0.118 ± 0.005
10	4.47	1.000	14.88	0.3	0.3	0.4	(3.8 ± 0.2) × 10^−4^	0.180 ± 0.006
11	5.07	1.000	15.15	0.1	0.1	0.2	(6.6 ± 0.7) × 10^−4^	0.32 ± 0.04

**Table 4:** CFZ solubility with increasing concentrations of malic acid in water at 25 °C. Experimental details, pH values, and solubilities expressed as molar and mg/mL concentrations are also given with standard deviations obtained from three measurements.

Vial Number	Acid Concentration (M)	Volume Used (mL)	CFZ Added (mg)	pH			CFZ Solubility (M)	CFZ Solubility (mg/mL)
				Before CFZ Addition	Before Phase Separation	After Phase Separation		
1	1.11 × 10^−2^	1.002	14.60	4.1	4.4	4.8	(2.31 ± 0.03) × 10^−5^	(1.09 ± 0.02) × 10^−2^
2	1.55 × 10^−2^	1.003	15.30	3.6	4.0	4.1	(2.1 ± 0.2) × 10^−5^	(1.01 ± 0.01) × 10^−2^
3	2.84 × 10^−2^	1.005	15.44	3.1	3.1	3.1	(2.7 ± 0.1) × 10^−5^	(1.27 ± 0.02) × 10^−2^
4	6.04 × 10^−2^	1.006	15.43	2.6	2.6	2.7	(2.83 ± 0.08) × 10^−5^	(1.34 ± 0.02) × 10^−2^
5	0.500	1.000	15.39	1.9	1.9	1.8	(1.91 ± 0.06) × 10^−5^	(9.1 ± 0.3) × 10^−3^
6	1.01	1.000	14.64	1.6	1.6	1.6	(1.34 ± 0.05) × 10^−4^	(6.3 ± 0.3) × 10^−2^
7	2.00	1.000	15.59	1.3	1.4	1.3	(3.2 ± 0.2) × 10^−4^	0.151 ± 0.005
8	3.01	1.000	15.39	1.1	1.1	1.1	(1.01 ± 0.05) × 10^−3^	0.48 ± 0.03
9	4.03	1.000	14.59	0.8	0.8	0.8	(1.36 ± 0.07) × 10^−3^	0.64 ± 0.04
10	4.54	1.000	14.48	0.7	0.7	0.7	(1.31 ± 0.05) × 10^−3^	0.62 ± 0.03
11	5.15	1.000	14.64	0.5	0.5	0.5	(2.59 ± 0.1) × 10^−3^	1.23 ± 0.05

**Table 5:** CFZ solubility with increasing concentrations of glutaric acid in water at 25 °C. Experimental details, pH values, and solubilities expressed as molar and mg/mL concentrations are also given with standard deviations obtained from three measurements.

Vial Number	Acid Concentration (M)	Volume Used (mL)	CFZ Added (mg)	pH			CFZ Solubility (M)	CFZ Solubility (mg/mL)
				Before CFZ Addition	Before Phase Separation	After Phase Separation		
1	1.28 × 10^−2^	1.003	14.87	4.1	4.5	4.6	(6.43 ± 0.02) × 10^−6^	(3.05 ± 0.07) × 10^−3^
2	1.74 × 10^−2^	1.003	15.50	3.6	3.8	3.8	(1.39 ± 0.02) × 10^−5^	(6.60 ± 0.09) × 10^−3^
3	3.04 × 10^−2^	1.002	14.79	3.1	3.3	3.4	(2.11 ± 0.06) × 10^−5^	(1.00 ± 0.03) × 10^−2^
4	0.232	1.002	15.22	2.5	2.8	2.8	(2.16 ± 0.05) × 10^−5^	(1.02 ± 0.03) × 10^−2^
5	0.494	1.000	15.39	2.3	2.4	2.4	(2.80 ± 0.04) × 10^−5^	(1.32 ± 0.02) × 10^−2^
6	1.01	1.000	14.95	2.1	2.2	2.2	(9.4 ± 0.3) × 10^−5^	(4.5 ± 0.2) × 10^−2^
7	2.00	1.000	15.40	1.9	1.9	2.0	(7.4 ± 0.2) × 10^−4^	0.350 ± 0.01
8	3.01	1.000	14.84	1.6	1.8	1.8	(3.1 ± 0.1) × 10^−3^	1.47 ± 0.05
9	4.04	1.000	52.62	1.4	1.6	1.6	(8.4 ± 0.3) × 10^−3^	4.0 ± 0.2
10	4.50	1.000	77.31	1.3	1.6	1.6	(1.36 ± 0.09) × 10^−2^	6.5 ± 0.4
11	5.06	1.000	73.91	1,1	1.5	1.5	(2.3 ± 0.1) × 10^−2^	10.7 ± 0.5

**Table 6:** Melting points of neat CFZ, studied weak acids and new crystalline materials formed after CFZ interaction with studied acids.

Sample	Melting Points of the New Crystalline Solids (°C)	Melting Points of Corresponding Weak Acids (°C)
CFZ	224.8	-
CFZ-Glutarate	77.0	99.8
CFZ-Citrate	207.6	157.2
CFZ-Tartarate	241.8	173.1
CFZ-Malate	248.5	134.1
CFZ-Maleic acid	220.9; 231.8	145.5
CFZ-Succinate	220.3	190.8
CFZ-Adipate	212.4	153.8

## Data Availability

Data will be made available on request.

## Supplementary Materials

The following supporting information can be downloaded at https://www.mdpi.com/article/10.3390/pharmaceutics16121545/s1, Table S1: Clofazimine (CFZ) solubility in solutions of 2 M and 5 M tartaric acid in water at 25 °C. Experimental details, stirring time, pH values and solubilities expressed as molar and mg/mL concentrations are also given; Table S2: Clofazimine solubility in solutions of 2 M and 5 M malic acid in water at 25 °C. Experimental details, stirring time, pH values and solubilities expressed as molar and mg/mL concentrations are also given; Table S3: Clofazimine solubility in solutions of 2 M and 5 M glutaric acid in water at 25 °C. Experimental details, stirring time, pH values and solubilities expressed as molar and mg/mL concentrations are also given; Figure S1: HPLC Chromatograms of CFZ external standard solutions; Figure S2: Calibration diagram for CFZ.
