# Supplementary material for: Effects of Different Weak Small Organic Acids on Clofazimine Solubility in Aqueous Media
Source: Pharmaceutics. 2024 Dec 2;16(12):1545. doi: 10.3390/pharmaceutics16121545 (PMC11728605; doi:10.3390/pharmaceutics16121545)
Supplement: Supplementary file 1 [file pharmaceutics-16-01545-s001.zip › pharmaceutics-3277398-supplementary.pdf]

## Supporting Information

# Effects of Different Weak Small Organic Acids on Clofazimine Solubility in Aqueous Media

**Igor A. Topalović [1], Olivera S. Marković [2], Miloš P. Pešić [1], Mufaddal H. Kathawala [3], Martin Kuentz [4], Alex Avdeef [5], Abu T. M. Serajuddin [3,*] and Tatjana Ž. Verbić [1,*]**

1 University of Belgrade – Faculty of Chemistry, Studentski trg 12-16, 11000 Belgrade, Republic of Serbia; itopalovic22@gmail.com; mpesic@chem.bg.ac.rs; tatjanad@chem.bg.ac.rs
2 University of Belgrade – Institute of Chemistry, Technology and Metallurgy, Department of Chemistry, Njegoševa 12, 11000 Belgrade, Republic of Serbia; olivera.markovic@ihtm.bg.ac.rs
3 St. John's University, College of Pharmacy and Health Sciences, Queens, New York 11439, USA; mufaddal.kathawala17@my.stjohns.edu; serajuda@stjohns.edu
4 University of Applied Sciences and Arts Northwestern Switzerland, School of Life Sciences FHNW, Institute for Pharma Technology, Hofackerstraße 30, 4132 Müttenz, Switzerland; martin.kuentz@fhnw.ch
5 in-ADME Research, New York, New York 10128, USA; alex@in-adme.com
* Correspondence: T.Ž.V. tatjanad@chem.bg.ac.rs (+381-63-255-356); A.T.M.S. serajuda@stjohns.edu

## CONTENTS

**Table S1**. Clofazimine (CFZ) solubility in solutions of 2 M and 5 M tartaric acid in water at 25°C. Experimental details, stirring time, pH values and solubilities expressed as molar and mg/mL concentrations are also given. 2

**Table S2**. Clofazimine (CFZ) solubility in solutions of 2 M and 5 M malic acid in water at 25°C. Experimental details, stirring time, pH values and solubilities expressed as molar and mg/mL concentrations are also given. 3

**Table S3**. Clofazimine (CFZ) solubility in solutions of 2 M and 5 M glutaric acid in water at 25°C. Experimental details, stirring time, pH values and solubilities expressed as molar and mg/mL concentrations are also given. 4

**Figure S1.** HPLC Chromatograms of CFZ external standard solutions 5

**Figure S2.** Calibration diagram for CFZ 6

**Table S1**. Clofazimine (CFZ) solubility in solutions of 2 M and 5 M tartaric acid in water at 25°C. Experimental details, stirring time, pH values and solubilities expressed as molar and mg/mL concentrations are also given.

| Vial | *c*(acid) (M) | *V* (mL) | *m*(Cfz) (mg) | Stirring time (h) | pH | | | *S*(Cfz) (M) | *S*(Cfz) (mg/mL) |
|---|---|---|---|---|---|---|---|---|---|
| | | | | | Before CFZ addition | Before phase separation | After phase separation | | |
| 1 | 2.00 | 1.000 | 14.98 | 1 | 1.0 | 0.9 | 0.8 | $5.5\times10^{-4}$ | 0.26 |
| 2 | 2.00 | 1.000 | 14.81 | 2 | 1.0 | 1.0 | 0.9 | $4.6\times10^{-4}$ | 0.22 |
| 3 | 2.00 | 1.000 | 14.15 | 3 | 1.0 | 1.0 | 1.0 | $5.1\times10^{-4}$ | 0.24 |
| 4 | 2.00 | 1.000 | 15.97 | 4 | 1.0 | 1.0 | 1.0 | $5.0\times10^{-4}$ | 0.24 |
| 5 | 2.00 | 1.000 | 14.87 | 5 | 1.0 | 1.1 | 0.9 | $4.7\times10^{-4}$ | 0.22 |
| 6 | 2.00 | 1.000 | 15.03 | 6 | 1.0 | 1.0 | 1.0 | $6.4\times10^{-5}$ | 0.03 |
| 7 | 2.00 | 1.000 | 14.38 | 6+18 [a] | 1.0 | 1.1 | 1.1 | $8.0\times10^{-5}$ | 0.04 |
| 8 | 5.00 | 1.000 | 15.58 | 1 | 0.2 | 0.2 | 0.1 | $5.9\times10^{-4}$ | 0.28 |
| 9 | 5.00 | 1.000 | 15.11 | 2 | 0.2 | 0.2 | 0.1 | $5.3\times10^{-4}$ | 0.25 |
| 10 | 5.00 | 1.000 | 15.89 | 3 | 0.2 | 0.2 | 0.2 | $5.3\times10^{-4}$ | 0.25 |
| 11 | 5.00 | 1.000 | 14.85 | 4 | 0.2 | 0.2 | 0.2 | $5.1\times10^{-4}$ | 0.24 |
| 12 | 5.00 | 1.000 | 14.58 | 5 | 0.2 | 0.2 | 0.2 | $6.3\times10^{-4}$ | 0.30 |
| 13 | 5.00 | 1.000 | 15.94 | 6 | 0.2 | 0.3 | 0.2 | $5.5\times10^{-4}$ | 0.26 |
| 14 | 5.00 | 1.000 | 15.02 | 6+18 [a] | 0.2 | 0.3 | 0.2 | $4.9\times10^{-4}$ | 0.23 |

[a] Stirring time + sedimentation time.

**Table S2**. Clofazimine (CFZ) solubility in solutions of 2 M and 5 M malic acid in water at 25°C. Experimental details, stirring time, pH values and solubilities expressed as molar and mg/mL concentrations are also given.

| Vial | *c*(acid) (M) | *V* (mL) | *m*(CFZ) (mg) | Stirring time (h) | pH | | | *S*(CFZ) (M) | *S*(CFZ) (mg/mL) |
|---|---|---|---|---|---|---|---|---|---|
| | | | | | Before CFZ addition | Before phase separation | After phase separation | | |
| 1 | 2.00 | 1.000 | 15.91 | 1 | 1.4 | 1.4 | 1.4 | $1.8\times10^{-4}$ | 0.08 |
| 2 | 2.00 | 1.000 | 15.98 | 2 | 1.4 | 1.3 | 1.3 | $3.2\times10^{-4}$ | 0.15 |
| 3 | 2.00 | 1.000 | 15.95 | 3 | 1.4 | 1.4 | 1.4 | $2.5\times10^{-4}$ | 0.12 |
| 4 | 2.00 | 1.000 | 14.33 | 4 | 1.4 | 1.4 | 1.4 | $3.3\times10^{-4}$ | 0.16 |
| 5 | 2.00 | 1.000 | 14.43 | 5 | 1.4 | 1.4 | 1.4 | $3.0\times10^{-4}$ | 0.14 |
| 6 | 2.00 | 1.000 | 14.32 | 6 | 1.4 | 1.4 | 1.4 | $2.1\times10^{-4}$ | 0.10 |
| 7 | 2.00 | 1.000 | 14.27 | 6+18 [a] | 1.4 | 1.4 | 1.4 | $1.7\times10^{-4}$ | 0.08 |
| 8 | 5.00 | 1.000 | 15.19 | 1 | 0.6 | 0.6 | 0.5 | $6.2\times10^{-3}$ | 2.9 |
| 9 | 5.00 | 1.000 | 14.53 | 2 | 0.6 | 0.7 | 0.6 | $3.7\times10^{-3}$ | 1.8 |
| 10 | 5.00 | 1,000 | 15.85 | 3 | 0.6 | 0.7 | 0.6 | $3.9\times10^{-3}$ | 1.9 |
| 11 | 5.00 | 1.000 | 15.77 | 4 | 0.6 | 0.8 | 0.6 | $3.8\times10^{-3}$ | 1.8 |
| 12 | 5.00 | 1.000 | 15.61 | 5 | 0.6 | 0.7 | 0.6 | $3.9\times10^{-3}$ | 1.9 |
| 13 | 5.00 | 1.000 | 15.90 | 6 | 0.6 | 0.6 | 0.6 | $4.0\times10^{-3}$ | 1.9 |
| 14 | 5.00 | 1.000 | 15.89 | 6+18[a] | 0.6 | 0.6 | 0.5 | $3.2\times10^{-3}$ | 1.5 |

[a] Stirring time + sedimentation time.

**Table S3**. Clofazimine (CFZ) solubility in solutions of 2 M and 5 M glutaric acid in water at 25°C. Experimental details, stirring time, pH values and solubilities expressed as molar and mg/mL concentrations are also given.

| Vial | *c*(acid) (M) | *V* (mL) | *m*(CFZ) (mg) | Stirring time (h) | pH Before CFZ addition | pH Before phase separation | pH After phase separation | *S*(CFZ) (M) | *S*(CFZ) (mg/mL) |
|---|---|---|---|---|---|---|---|---|---|
| 1 | 2.00 | 1.000 | 15.50 | 1 | 1.9 | 1.9 | 1.7 | $6.2\times10^{-4}$ | 0.29 |
| 2 | 2.00 | 1.000 | 15.09 | 2 | 1.9 | 1.9 | 1.8 | $5.4\times10^{-4}$ | 0.25 |
| 3 | 2.00 | 1.000 | 15.87 | 3 | 1.9 | 1.9 | 1.9 | $5.6\times10^{-4}$ | 0.26 |
| 4 | 2.00 | 1.000 | 14.57 | 4 | 1.9 | 1.9 | 1.9 | $5.7\times10^{-4}$ | 0.27 |
| 5 | 2.00 | 1.000 | 15.22 | 5 | 1.9 | 1.9 | 1.9 | $5.5\times10^{-4}$ | 0.26 |
| 6 | 2.00 | 1.000 | 14.42 | 6 | 1.9 | 1.9 | 2.0 | $6.0\times10^{-4}$ | 0.28 |
| 7 | 2.00 | 1.000 | 14.96 | 6+18 [a] | 1.9 | 1.9 | 1.9 | $6.5\times10^{-4}$ | 0.32 |
| 8 | 5.00 | 1.000 | 50.66 | 1 | 1.2 | 1.5 | 1.5 | $2.0\times10^{-2}$ | 9.5 |
| 9 | 5.00 | 1.000 | 49.38 | 2 | 1.2 | 1.5 | 1.5 | $1.8\times10^{-2}$ | 8.6 |
| 10 | 5.00 | 1.000 | 49.37 | 3 | 1.2 | 1.5 | 1.5 | $1.8\times10^{-2}$ | 8.7 |
| 11 | 5.00 | 1.000 | 50.53 | 4 | 1.2 | 1.5 | 1.5 | $1.9\times10^{-2}$ | 9.1 |
| 12 | 5.00 | 1.000 | 50.25 | 5 | 1.2 | 1.5 | 1.5 | $1.9\times10^{-2}$ | 8.9 |
| 13 | 5.00 | 1.000 | 50.91 | 6 | 1.2 | 1.5 | 1.6 | $2.0\times10^{-2}$ | 9.4 |
| 14 | 5.00 | 1.000 | 50.95 | 6+18 [a] | 1.2 | 1.5 | 1.5 | $1.9\times10^{-2}$ | 9.1 |

[a] Stirring time + sedimentation time.

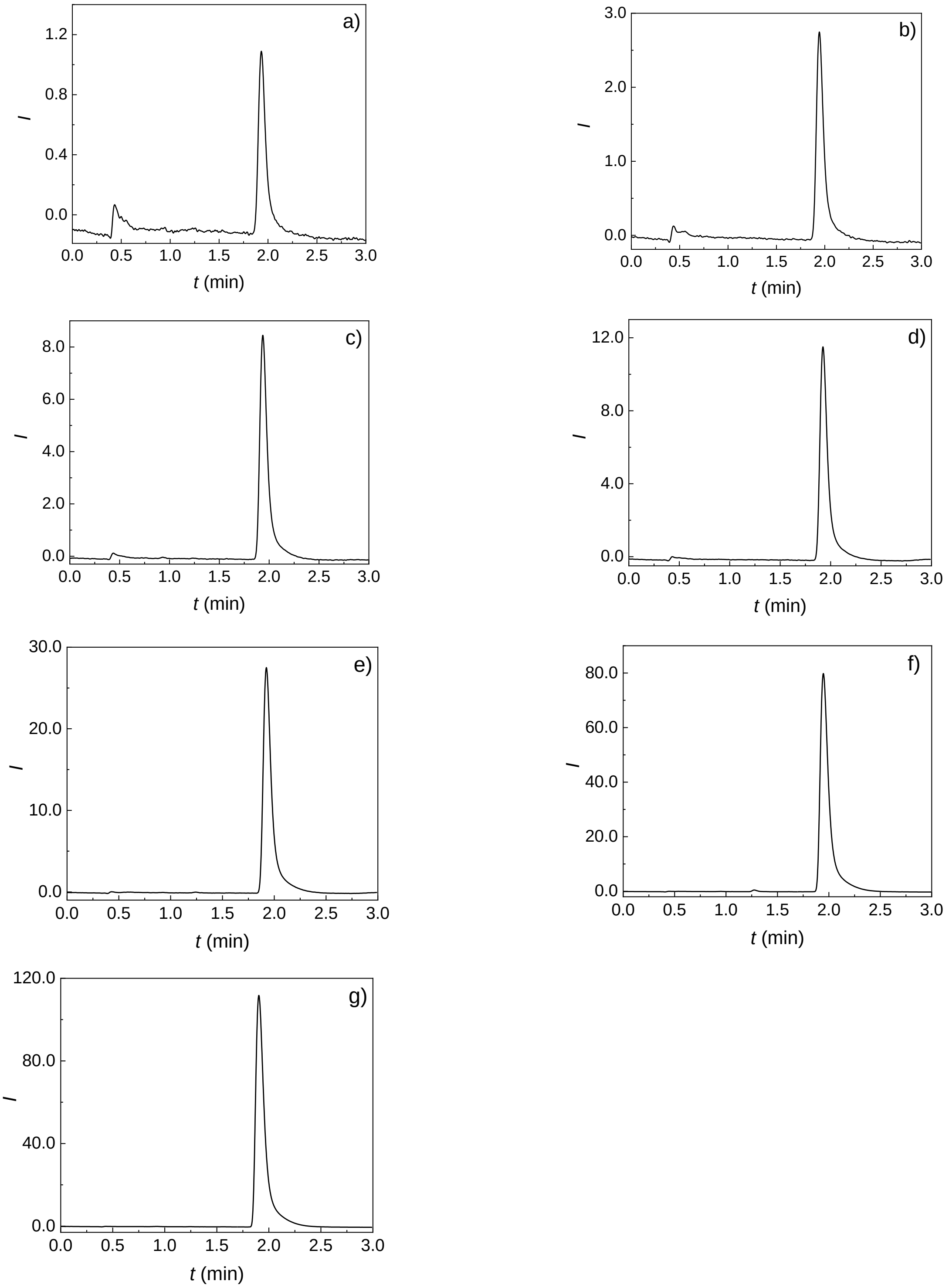


**Figure S1.** HPLC Chromatograms of CFZ external standard solutions with following concentrations: **a**) $1.00\times10^{-6}$ M, **b**) $2.50\times10^{-6}$ M, **c**) $7.50\times10^{-6}$ M, **d**) $1.00\times10^{-5}$ M, **e**) $2.50\times10^{-5}$ M, **f**) $7.50\times10^{-5}$ M, **g**) $1.00\times10^{-4}$ M.

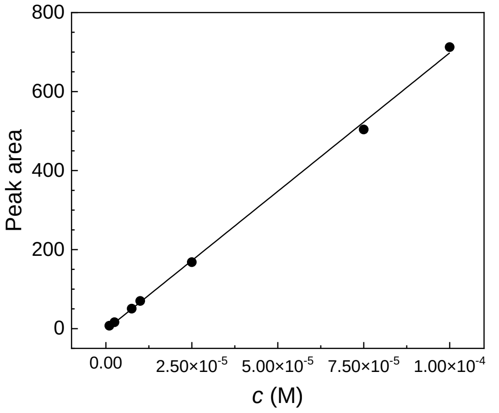


**Figure S2.** Calibration diagram for CFZ (concentration range $1.00\times10^{-6}$ – $1.00\times10^{-4}$ M) Linear fit: Peak area = $7.01\times10^{6} \times c - 2.83$ ($r^2 = 0.998$)
